# Inherited Diseases

**Published:** 1867-12

**Authors:** Wm. O. Kulp

**Affiliations:** Muscatine, Iowa


### INHERITED DISEASES.

In reading the short article by Dr. Chase on "inherited diseases," and the "W. editorial comment on it," I am forcibly impressed with a fact or two that occurred in my own practice. Mrs. H., a newly married lady, came to my office at the urgent suggestion of her adoring husband, to consult me about the possibility of smoothing the surface of a pitted lower left central incisor tooth, which looked rather unsightly and seemed to annoy her husband greatly. I decided to dress the tooth down and polish it, which was done without causing much annoyance; a great deal of satisfaction was expressed at the appearance of the tooth after the operation. I heard no more of the case for about seven months, when the husband and now *father* came hurriedly into my office, stating that "*the baby*" had a tooth, wished me to call at his house and examine it, as he feared it was something that might cause trouble. I called in a few days, and on examination found that it was a lower left central incisor tooth, a fac-simile of the one I had seven months before polished for the mother; it had the same pitted appearance.

CASE 2.—Mrs. A., a very robust lady, the mother of three children, called to have some teeth filled. I filled some eight or ten cavities, four of which were very sensitive, and teeth considerably broken off; the crowns were restored with gold, the operation was quite lengthy and painful. About eight months subsequent she gave birth to a healthy boy with four teeth corresponding with those with the large gold fillings in her own mouth. These cases created quite a sensation in their immediate neighborhoods.

I have followed up the advantages thus providentially opened, and try to impress the fact upon the minds of parents that they have the power to transmit *any* physiological condition to their offspring they choose, and trust I will see some good results in the course of time. While I am on this subject, I will state a case or two of a little different character; but I hope of no less interest to the profession. By consulting mothers who have had several children, we will find that they have universally more trouble with their teeth during gestation, and more especially during lactation, than any other period of life.

Mrs. —, consulted me frequently during her first pregnancy, and afterwards during lactation, as she seemed to suffer greatly with her teeth. I would find her teeth very sensitive to thermal changes, and to acids and saccharine substances, and towards the later period of lactation I found her teeth very soft and almost devoid of their earthy substances. In the course of time she became pregnant again, and the old trouble with the teeth began again. I advised a diet of food containing the greatest amount of the phosphates of lime. I heard but little more of the teeth. I would examine the teeth occasionally, and I found them hard and pearly, and not at all sensitive as before; and in due time she gave birth to a very much larger and more robust child than she had before and with less labor than formerly, and her teeth continued the same pearls during lactation; her child, now three years old, has the most perfect set of deciduous teeth I ever saw, as firm and solid as adamant; the mother is still following the dietetic suggestions of four years ago, and has suggested it to many others with equally good and satisfactory results. What better evidence do we want, that teeth of *adults* as well as in the embryonic or infantile state can be nourished. WM. O. KULP, D. D. S.

MUSCATINE, Iowa, Dec. 20th, 1867.

